# Male responses to sperm competition risk associated with increased macronutrient intake and reduced lifespan

**DOI:** 10.1098/rsbl.2023.0336

**Published:** 2023-10-25

**Authors:** Leigh W. Simmons, Hwei-Ling Chan

**Affiliations:** Centre for Evolutionary Biology, School of Biological Sciences, The University of Western Australia, Crawley 6009, Australia

**Keywords:** sperm competition risk, nutritional ecology, life-history trade-off, strategic ejaculation, lifespan

## Abstract

Increased expenditure on the ejaculate is a taxonomically widespread male response to sperm competition. Increased ejaculate expenditure is assumed to come at a cost to future reproduction, otherwise males should always invest maximally. However, the life-history costs of strategic ejaculation are not well documented. Macronutrient intake is known to affect the trade-off between reproduction and lifespan. Intakes of protein and carbohydrate that maximize reproduction often differ from those that maximize lifespan. Here, we asked whether strategic expenditure on the ejaculate by male crickets, *Teleogryllus oceanicus*, is mediated by macronutrient intake, and whether it comes at a cost of reduced lifespan. Males were exposed to rival song throughout their lifespan or were held in a silent non-competitive environment. Males exposed to song had a higher intake of both protein and carbohydrate, they reached adulthood sooner, produced ejaculates of higher quality, and died sooner than males living in a silent environment. Our findings provide a rare example of both the mechanisms and life-history costs associated with strategic ejaculation.

## 1. Introduction

Strategic ejaculation in response to variation in the competitive environment is a taxonomically widespread phenomenon; the males of both invertebrate and vertebrate species have been found to increase their expenditure on ejaculate production when reared in a competitive environment, and to allocate greater quantities of sperm and seminal fluid to a given mating when the immediate risk of sperm competition is elevated [1–3]. It is generally assumed that increased male expenditure on and allocation of ejaculate components is adaptive, in that it should increase a male's competitive fertilization success, and that ejaculate expenditure must be costly, otherwise one would expect males to invest maximally at each mating opportunity [4–6]. However, the costs and benefits of strategic ejaculation are less well studied than the phenomenon itself [3].

Studies of *Drosophila* have found that increased allocation to the ejaculate can increase a male's immediate reproductive returns [7–10]. The same is true of soldier flies, *Merosargus cingulatus* [11]. However, no increase in fitness was associated with male responses to rivals in burying beetles, *Nicrophorus vespilloides* [12] and in mosquitofish, *Gambusia holbrooki*, males exposed to rival males had slower-swimming sperm and reduced paternity compared with males from a control group that had not been exposed to rivals [13]. The long-term life-history costs of male strategic responses to rivals could provide an explanation for the later, apparently counterintuitive finding. The fish were held in their competitive treatments for five weeks before being assayed, and prolonged exposure and response to rivals could have reduced male fitness when they were finally allowed to mate [13]. However, the costs associated with strategic ejaculation are even less well studied than the benefits [3,14]. Attempts to establish the costs of strategic ejaculation within a life-history context are limited to three studies of *Drosophila*. Increased ejaculate expenditure in response to rivals was found to increase short-term reproductive success, but at a cost to both late-life mating success and lifespan [8,15,16].

Macronutrient intake is widely known to impact an organisms life history. In general, expenditure on reproduction tends to be maximized by a higher intake of protein relative to carbohydrate while lifespan is maximized by a lower intake of protein relative to carbohydrate, providing a nutritional mechanism for the well-established life-history trade-off between reproduction and lifespan [17,18]. Ejaculates are known to be costly for males to produce, and particularly sensitive to nutrition [19]. Sperm production and fertility are maximized in house mice by diets with a high protein-to-carbohydrate (P : C) ratio [18], while in *Drosophila* and the cockroach *Nauphoeta cinerea*, sperm numbers and fertility are maximized by diets with a low P : C ratio [20]. In all cases, the P : C ratio that maximizes ejaculate production was found to be associated with reduced lifespan. Given that animals are capable of actively adjusting their intake of macronutrients in such a way as to optimize fitness [21–23], one potential mechanism for strategic allocation to ejaculate production could be for males to adjust their macronutrient intake target in response to rival males, at the life-history cost of reduced lifespan. Here, we test this possibility using the field cricket *Teleogryllus oceanicus*.

In *T. oceanicus* sperm production and sperm viability are both maximized by a high intake of micronutrients and a low P : C ratio, while lifespan is maximized by a low intake of micronutrients and a more balanced P : C ratio [24]. Sperm viability is a key determinant of a male's competitive fertilization success [25], and male *T. oceanicus* exposed to the acoustic signals of rival males have been found to increase their expenditure on testes and accessory gland growth [26], and the seminal fluid composition and viability of sperm contained within their ejaculates [27–29]. Here, we exposed males to the songs of rivals or raised them in silence while allowing them to feed freely on artificial diets so as to track their nutrient intake. We monitored crickets for their entire lifespan, and measured their sperm viability at early and mid-adult life. We predicted that males exposed to rival songs should adjust their nutrient intake to produce ejaculates of superior quality, and that upregulation of ejaculate quality in response to rivals should come at the cost of reduced lifespan.

## 2. Material and methods

Crickets used in this study were drawn from a laboratory stock that originated from Carnarvon in Western Australia, and was kept as a large outbreeding population (greater than 1000 individuals) supplemented with wild caught crickets annually. Crickets were maintained at 26°C under a 12 h light : 12 h dark cycle and fed cat chow as the routine diet. Experimental crickets were drawn from this stock population at the 8th instar, when sex can first be determined.

Male crickets were isolated in individual plastic containers (7 × 7 × 5 cm) and provided with ad libitum access to water via a cotton plugged vial, and a choice of two chemically defined diets containing either protein or carbohydrate [30]. Crickets were split equally across two competitive environments, one in which they were exposed to the acoustic sexual signals of rival males (song = 38) and the other a non-competitive silent environment (silence = 38). Manipulation of the competitive environment followed established protocols [31,32], details of which can be found in the electronic supplementary material. Crickets were checked daily, and the date on which they moulted to the 9th (penultimate) instar, and the date they moulted to an adult were noted.

Crickets were weighed on the day they moulted to adulthood, and silenced by clipping the file and plectrum from the sound-producing forewings. Silenced males ‘stridulate’ normally as adults, but the lack of a file and plectrum prevents any sound being produced. Nutrient intake was then monitored until their day of death. Two small dishes, one containing the protein diet and the other the carbohydrate diet, were placed into each male's container. Diet composition and preparation followed established protocols [30], details of which can be found in the electronic supplementary material. Dishes were loaded with diet that had been dried in an oven stocked with packets of silica gel desiccant at 30°C for 24 h. Food dishes were exchanged every Monday, Wednesday and Friday. Fresh dishes were weighed before being placed into a cricket's container. Used dishes were removed and dried as previously before being weighed again to determine the amount of each diet consumed.

We measured five life-history traits, the duration of the penultimate instar (days between 9th instar and adult moults), weight at adult moult, sperm viability in early (14 days) and mid (28 days) adult life and lifespan (days from adult moult to death). Sperm viability was estimated as the proportion of live sperm in the ejaculate following established protocols [29] (for details, see the electronic supplementary material). Total macronutrient intake was calculated as the sum of protein or carbohydrate diet consumed in grams per gram of cricket per day of life, multiplied by 0.42 (the macronutrient concentration in the chemically defined diets, see electronic supplementary material). All statistical analyses were conducted in the statistical software package JMP 15.2.0.

## 3. Results

**Figure 1.**
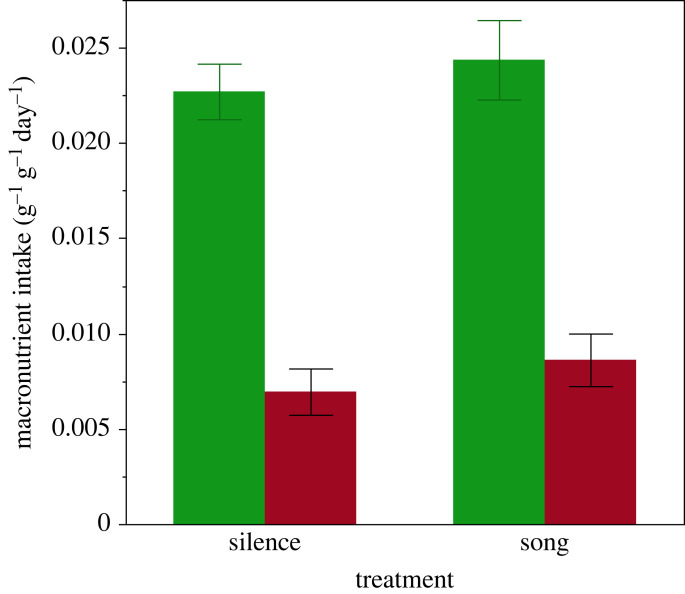
Total intake (± s.e.) of carbohydrate (green) and protein (red) by male crickets exposed to rival acoustic signals (song) or held in acoustic isolation (silence).

Repeated measures analysis of variance revealed a significant effect of competitive environment on macronutrient intake (between subject effect of treatment: *F*_1, 74_ = 4.40, *p* = 0.039), a significant within subject effect of macronutrient type (*F*_1, 74_ = 436.6, *p* < 0.001), but no significant macronutrient by treatment interaction (*F*_1, 74_ = 0.00, *p* = 0.996). Males consumed a greater amount of carbohydrate than protein, and increased their total intake of both macronutrients equally when exposed to rival song (figure 1). The intake ratio of P : C was 1 : 3.4 (95% CIs 1 : 2.9, 1 : 3.9).

Males exposed to rival songs had a shorter penultimate nymphal instar than those reared in silence, but treatment had no effect on weight at adult eclosion (table 1). Not all males produced a spermatophore at both days 14 and 28. There was no effect of treatment on the probability that males would produce a spermatophore (day 14, likelihood ratio *χ*^2^ = 1.90, d.f. = 1, *p* = 0.168; day 28: *χ*^2^ = 2.61, d.f. = 1, *p* = 0.106). Overall, spermatophores were available for 16 and 22 males in the song and silent treatments, respectively, at day 14, and for 17 and 24 males at day 28. Males exposed to rival song had greater sperm viability than did males held in the silent environment, significantly so at day 28 (table 1). Males exposed to rival song had a significantly shorter lifespan than males from the non-competitive silent environment (table 1).

**Table 1.** Life-history traits (mean [95% CI] (*n*)) of male *Teleogryllus oceanicus* exposed to the acoustic sexual signals of rivals (song) from the 8th instar and throughout adult life, compared with those of males held in acoustic isolation (silence).

	song	silence	F	d.f.	*p*-value
days to adult moult	11.1 [10.3, 11.9] (38)	13.4 [12.2, 14.5] (38)	9.41	1, 74	0.003
adult weight (mg)	0.44 [0.42, 0.47] (38)	0.43 [0.40, 0.45] (38)	1.18	1, 74	0.281
sperm viability day 14	0.58 [0.48, 0.68] (15)	0.49 [0.38, 0.59] (22)	1.56	1, 35	0.219
sperm viability day 28	0.70 [0.65, 0.76] (12)	0.54 [0.50, 0.57] (21)	30.90	1, 31	< 0.001
lifespan	58.5 [51.3, 65.7] (38)	74.4 [66.8, 82.1] (38)	9.41	1, 74	0.003

## 4. Discussion

Male crickets exposed to the calls of rivals were found to increase their total nutrient intake. They reached adulthood sooner and produced ejaculates with a greater proportion of viable sperm. However, they experienced a reduced lifespan compared with males living in a non-competitive, silent environment. These findings suggest that males suffer a life-history cost of reproduction that is associated with strategic increases in ejaculate expenditure.

Males did not adjust the ratio of P : C in their diets in response to rivals, rather they increased their total nutrient intake via both carbohydrate and protein diets. Consistent with previous studies males in both environments chose a macronutrient intake target of 1 : 3 P : C [23,24]. Diets with a high protein content have been found to be detrimental to sperm production in this species, and a P : C balance of 1 : 3 appears to optimize ejaculate production and sperm viability given the higher protein intakes that are required to produce attractive courtship songs and cuticular hydrocarbon profiles [24]. Increased intake of both protein and carbohydrate have been shown to increase testes and accessory gland mass, and to increase the expression of at least two seminal fluid proteins that affect sperm viability [33]. Moreover, increased dietary intake would also have resulted in an increase in total micronutrients which were available in fixed quantities in both diets. Increased micronutrient intake has been found to increase sperm viability [24]. Collectively, these studies suggest that the elevated sperm viability found for males exposed to rivals in the current study was likely due to their increased total dietary intake. Moreover, increased nutrient intake is known to decrease adult lifespan [24], which would have contributed to the reduced lifespan of males exposed to rivals found here. Thus, strategic adjustments in ejaculate expenditure by male *T. oceanicus* appear to be mediated in part via adjustments in nutrient intake and come at a cost of reduced lifespan.

Although sperm viability was higher for males exposed to rival song at both 14 and 28 days of adult age, the difference between treatments was statistically significant only when males were assayed at 28 days of age. Previously it was found that the protein content of seminal fluid in male *T. oceanicus* changes as males age, with the abundance of several seminal fluid proteins increasing up to 16 days after the adult moult [34]. These changes in seminal fluid protein composition are reflected in increased sperm viability [25] and competitive fertility [35] as males mature. Thus, a potential explanation for the non-significant difference in sperm viability between treatments when males were aged 14 days could be that males were not yet fully mature, and/or had not had sufficient time to fully adjust their ejaculates to the prevailing competitive environments. Moreover, males first encountered females when aged 14 days, so that prior sexual experience might also contribute to the greater effect when males were 28 days of age.

Males exposed to rival song also reached adult moult sooner than those reared in silence, indicative of competitive growth. Competitive growth in response to rivals has been reported previously, in both invertebrates [36] and vertebrates [37–39]. Collectively, our findings show how males can adjust their life history in response to competition, to one in which they live faster and die younger, and provide a rare example of the life-history costs associated with strategic ejaculation.

## Data Availability

The data required to reproduce the analyses reported in this paper are available from the Dryad Digital Repository: https://doi.org/10.5061/dryad.kwh70rz9j [40]. Extended methods are provided in the electronic supplementary material [41].
